# Topographical Anisotropy and Wetting of Ground Stainless Steel Surfaces

**DOI:** 10.3390/ma5122773

**Published:** 2012-12-12

**Authors:** Alfredo Calvimontes, Marc Mauermann, Cornelia Bellmann

**Affiliations:** 1 Leibniz Institute of Polymer Research Dresden, Hohe Strasse 6, 01069 Dresden, Germany; E-Mail: bellmann@ipfdd.de; 2 Fraunhofer Application Centre for Processing Machines and Packaging Technology, Heidelberger Strasse 20, 01189 Dresden, Germany; E-Mail: marc.mauermann@avv.fraunhofer.de

**Keywords:** topographical anisotropy, wetting, stainless steel surfaces, wetting regimes

## Abstract

Microscopic and physico-chemical methods were used for a comprehensive surface characterization of different mechanically modified stainless steel surfaces. The surfaces were analyzed using high-resolution confocal microscopy, resulting in detailed information about the topographic properties. In addition, static water contact angle measurements were carried out to characterize the surface heterogeneity of the samples. The effect of morphological anisotropy on water contact angle anisotropy was investigated. The correlation between topography and wetting was studied by means of a model of wetting proposed in the present work, that allows quantifying the air volume of the interface water drop-stainless steel surface.

## 1. Introduction

The quantitative description of the microstructure and the surface topography is a research field, which can provide a better understanding of the relation between surface topography, microstructure, and mechanical and physical-chemical properties. Surfaces of materials contain information about the mechanism of their formation as well as the factors that have influence on this mechanism. Besides, the surface morphology of a material can essentially influence its functional character. In many cases, a systematic surface characterization is necessary to set up quantitative correlations between production conditions and physico-chemical properties of engineering surfaces to compare the resulting surface with standards and to model surface behavior.

During the last 30 years, the possibilities for surface topography quantification have been broadened by the availability of new methods [1]. For the evaluation of topographical data, several mathematical operations such as calculation algorithms and standard parameters can be applied today [2]. For this reason, the selection of the correct methodology while evaluating the data measured and the optimal use of the topographical information obtained is especially relevant.

For any type of modification of a technical surface, the interplay between topography and surface chemistry determines the surface properties. Therefore, topographic qualitative description (morphology) and its quantitative description (topometry) are of great importance. Every modification can produce changes on the surface in a special way. Additional to the nature of the process (mechanical, optical, electrical, magnetic, chemical and biological), the duration of its effect and external mechanical/environmental influences must be considered in general [3]. The resulting topography correlates to nanoscopic, microscopic and macroscopic properties, which in combination define the final surface properties.

This paper is focuses on investigating the effect of morphological anisotropy on water contact angle. The correlation between topography and wetting of the grounded surfaces of stainless steel was investigated by means of a mathematical model, which is proposed in the present work to describe the wetting properties. In addition, this new model allows the calculation of the enclosed air volume in the interface between water and stainless steel. Recently, Ishino *et al.* [4] proposed a model to describe the transition states between metastable contacts and to quantify the energy barriers between them. With the use of phase diagrams in the two dimensional space of texture parameters they postulate transitional stages between the different wetting regimes. Further, Kioshi *et al.* [5] presented a simulation evidence of coexisting Wenzel/Cassie [6,7] state for water droplets on a pillared hydrophobic surface. According to their results, a critical pillar height exists beyond which water droplets on pillared hydrophobic surfaces can be in the bistable Wenzel/Cassie state, depending on the initial condition of the droplets. To reach these results, Kioshi *et al.* computed the free-energy barrier separating the Wenzel and Cassie states on the molecular level, based on a statistical mechanics method.

The model presented in this paper is a validation of that presented by Kioshi *et al* for the molecular scale, adapted to the microscopic scale.

## 2. Materials

An untreated stainless steel sample and six ground samples with different roughness values were used in this study (Table 1). The material examined was an austenitic stainless steel ANSI 316L 2B, which is notable for its face-centered cubic structure that improves the ductility and high corrosion resistance compared to plain carbon steels. Grinding damages the crystallite structures and dramatically changes the topography by giving the surface a defined anisotropy.

**Table 1:** Sample characteristics.

Sample Number	Grinding method	Abrasive paper CAMI grit designation	Abrasive paper average particle diameter (µm)
0	Cold-rolled, pickled	-	-
1	Ground and polished	600	16
2	Ground	600	16
3	Ground	360	28
4	Ground	240	53
5	Ground	150	92
6	Ground	80	190

## 3. Methods

### 3.1. Topographic Characterization

The topographic characterization was realized by means of high-resolution scandisk confocal microscopy (SDCM). This is an optical imaging technique used to increase micrograph contrast and/or to reconstruct three-dimensional images by using a spatial pinhole to eliminate out-of-focus light or flare in specimens that are thicker than the focal plane (United States Patent 6824056) [8]. This method allows a fast 3D measurement of topography, structure and roughness with excellent height resolution and depth of field. In this study, a µSurf (Nanofocus AG, Germany) device was used. To characterize the stainless steel samples, a cut-off length of *L_m_* = 260 µm, a lateral resolution of Δ*x* = 0.3 µm and a vertical resolution of Δ*z* = 2–6 nm was used. Four measurements on different positions were done on each sample.

### 3.2. Characterization of Wettability

**Figure 1:**
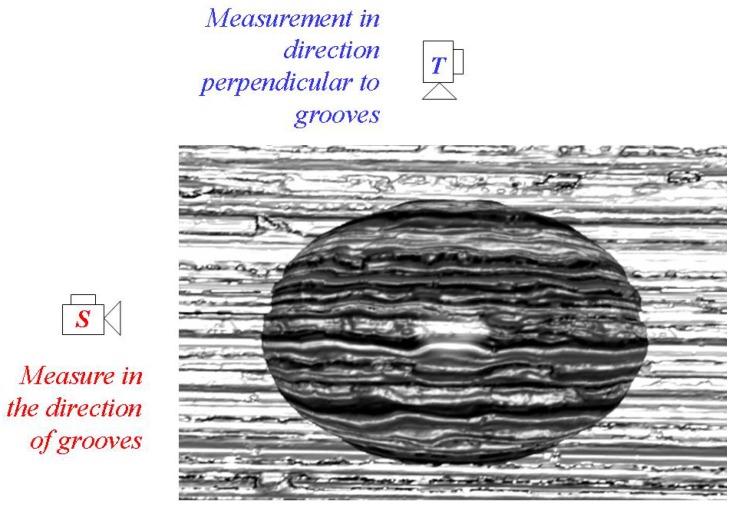
Schematic representation of the contact angle measurement from both directions, ***S*** and ***T***.

**Figure 2:**
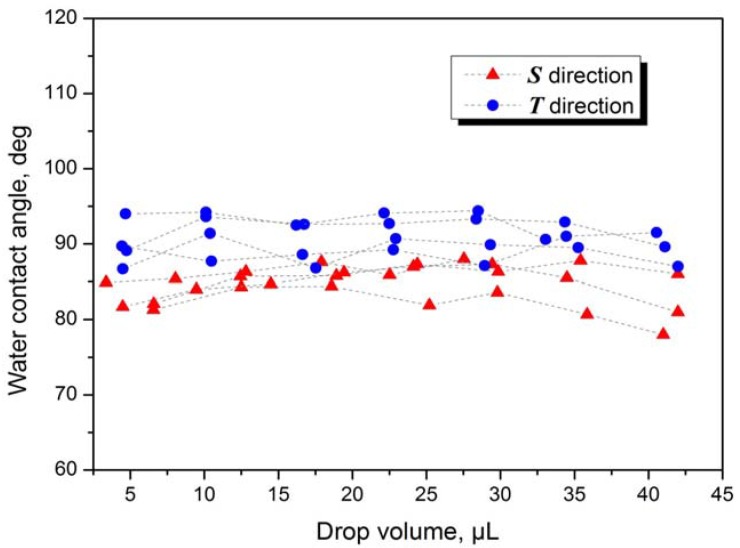
Gravitational effect of drop volume on contact angles for Sample 1.

Wettability was characterized by means of static contact angle measurements. To determine the static contact angle *θ*, a measuring device OCA 40 Micro (Data Physics, Germany) was used. Prior to the characterization of wetting, the effect of gravity on the water drop volume was investigated. For this purpose, different drop volumes were applied on Sample 1. The contact angles were observed in the sanding direction ***S*** of the stainless steel and perpendicular ***T*** to this direction (Figure 1). According to results shown in Figure 2, there was no significant effect of gravity for volumes equal or less than 30 µL. For this reason, 30 µL deionized water drops were used for the characterization of the wettability. The drops were placed on the surface with the help of a microliter syringe. Thereafter, the needle of the syringe was withdrawn from the drops and the contact angles were determined with the help of the software SCA 20th. Five water drops were applied to each specimen to determine the static contact angles observed in direction ***S*** and five measurements were made perpendicular ***T*** to this direction.

## 4. Results and Discussion

**Figure 3:**
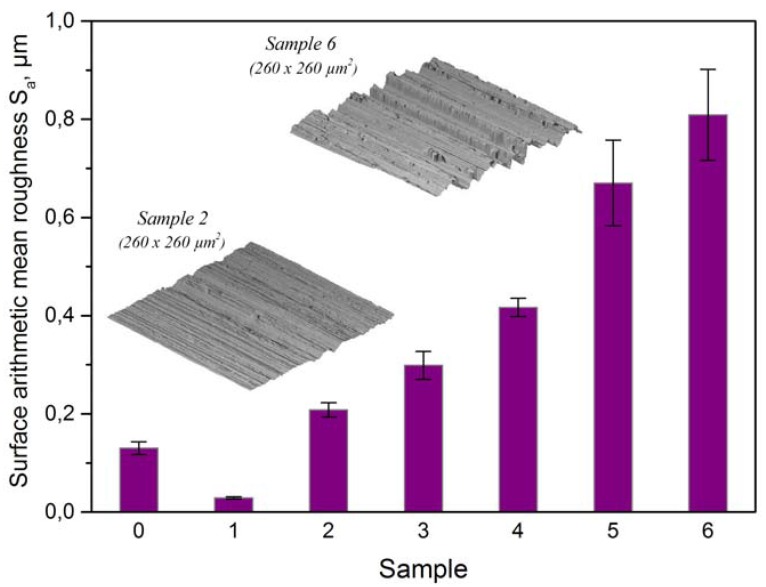
Surface arithmetic mean roughness (DIN EN ISO 25178) [9]. Error bars are the standard deviations.

Unidirectional machine grinding of untreated samples with different grains (Table 1) resulted in samples of six different topographies. These differences can be appreciated using the surface arithmetic mean roughness *S_a_* (DIN EN ISO 25178) [9], as shown in Figure 3. As will be shown later, for a more complete study of the topography, two additional parameters were used: the surface area ratio—also known as Wenzel factor—and the reduced roughness.

**Figure 4:**
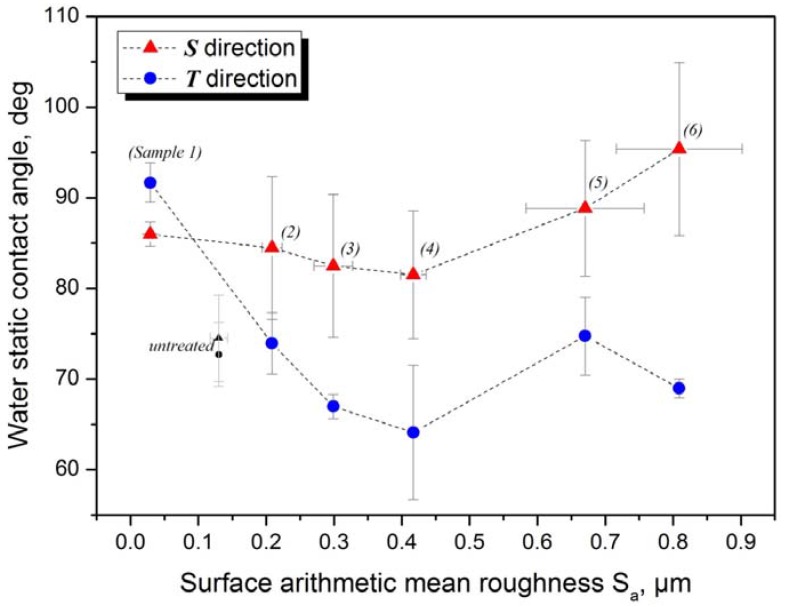
Dependence of static contact angle on the surface arithmetic mean roughness. Error bars are the standard deviations.

The dependence of the static contact angle on the direction of the measurement has previously been studied by Shuttleworth and Bailey [10], Chen *et al.* [11] and by Neuhaus *et al.* [12]. In the present study, all ground surfaces showed important differences between contact angles measured in ***T*** and ***S*** directions. This contact angle anisotropy is proportional to the surface arithmetic mean roughness *S_a_*, as shown in Figure 3. Rougher samples show larger wetting anisotropy than the smoother ones. The untreated sample, which is almost isotropic, shows the smallest difference between *θ*_T_ and *θ*_S_. Contact angles observed in ***T*** direction are, except in the case of Sample 1, smaller than those observed in ***S*** direction because grooves drive the water by capillary force. In both directions, minima are observed at the roughness value corresponding to Sample 4 (Figure 4).

**Figure 5:**
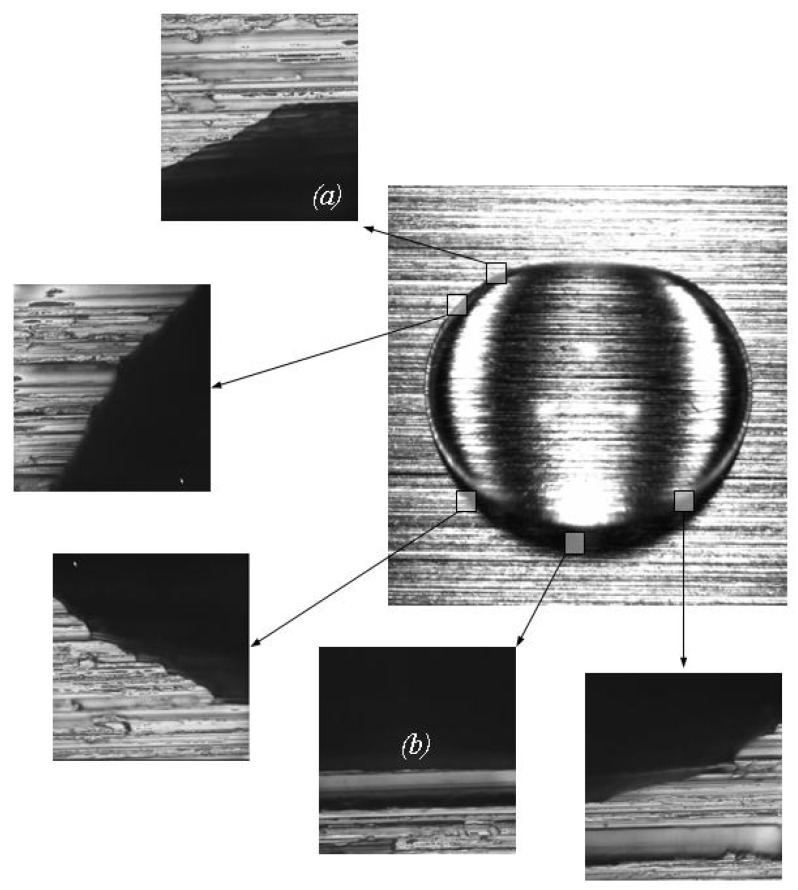
Effect of topographic anisotropy of stainless steel on the water drop boundary at different locations. The measure side length of the droplet image is 5 mm. The measured side length of all the zooms is 160 µm.

**Figure 6:**
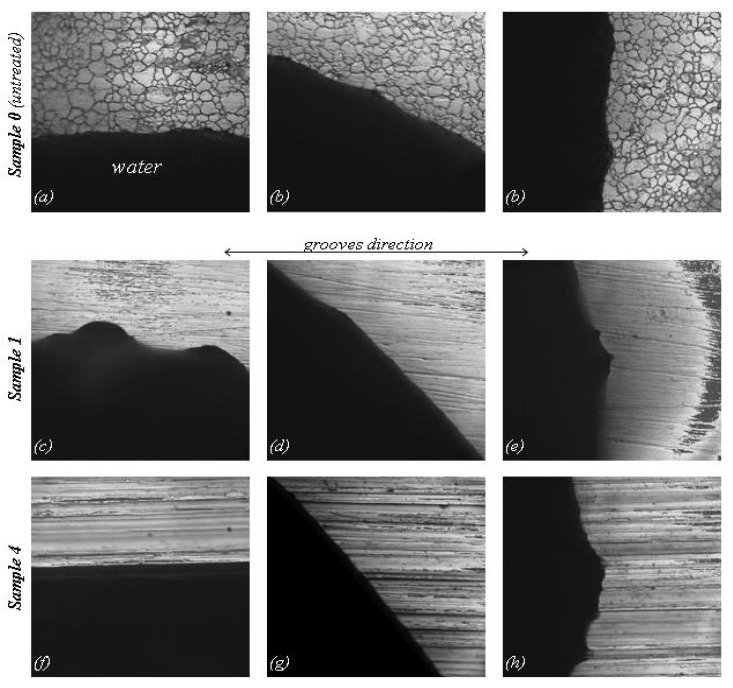
Effect of topographic anisotropy of stainless steel on the water drop boundary at different locations. The measured side length of all the images is 160µm

The microscopic grooves of stainless steel surface formed during grinding determine the geometry of the water droplet boundary. This effect of the topographic anisotropy can be seen by means of the confocal microscope images (Figure 5 and Figure 6). Water is detained in the places where it flows perpendicular to the groves (Figure 5a,b and Figure 6f), but tends to flow along the groves by capillary force (Figure 6h). On the intermediate contact lines, neither perpendicular nor parallel to the groves (Figure 6d,g), the water boundary is far more regular. Although the ground/polished Sample 1 is the least rough, its boundaries are not at all regular. Unlike the rest, water tends to spread in the direction perpendicular to the grooves (Figure 6c) resulting in a static contact angle of *θ***_T_** > *θ***_S_**, as shown in Figure 4.

**Figure 7:**
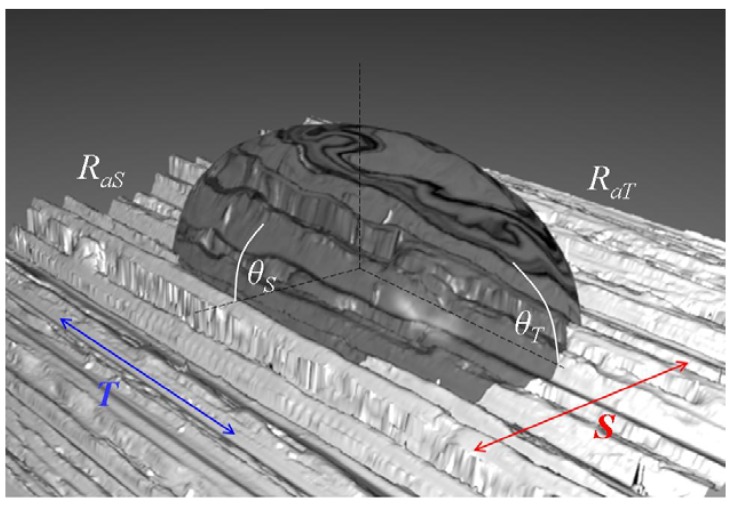
Schematic representation of contact angles *θ***_T_** and *θ***_S_**

**Figure 8:**
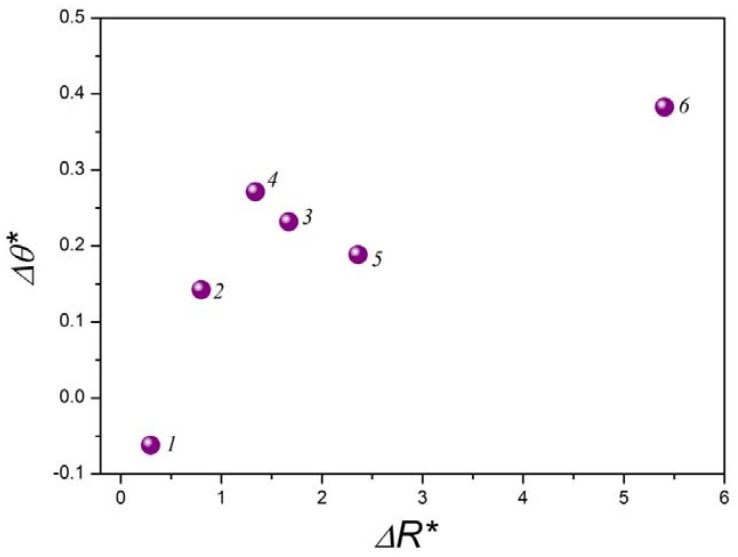
Relationship between topographical anisotropy and contact angle anisotropy using the T-S Model.

To better understand the effect of topography and surface anisotropy on wetting, we can decompose the geometry of the droplet into two orthogonal components ***T*** and ***S***, according to Figure 7. Using this model, it is possible to describe the effect of the topographical anisotropy on wetting by quantifying the topographical anisotropy by means of the reduced parameter ΔR* and the reduced contact angle anisotropy Δθ* (Figure 8):

(1) $$\Delta R*\;=\;\frac{R_{aS}\;-\;R_{aT}}{R_{aT}}$$

(2) $$\Delta \theta *\;=\;\frac{\theta_{S}\;-\;\theta_{T}}{\theta_{T}}$$

where *R_a**T**_* and *R_a**S**_* are the 2D-arithmetic roughness (DIN 4768, ASME B46.1) of the surface profiles measured in direction ***T*** and ***S***, respectively. The use of ΔR* and Δθ* is intended to measure the anisotropy by quantifying the differences between the parameters measured in both directions and relating these differences to the direction with the smoother topography, used as local reference. According to Figure 8 there is a significant relationship between the topographic anisotropy and wettability in both directions. Once we have proved this relationship, it is possible to use this ***S-T*** components model to investigate the wetting regime on these modified steel surfaces.

Considering that wetting is complete on the whole surface, Young’s equation could be used to calculate the contact angle of a rough, chemically homogeneous surface by using the roughness factor *r* introduced by Wenzel in 1936 [6] and defined as the ratio of the actual area of a rough surface to the geometric projected area on the horizontal plane:

(3) $$\cos\theta_{w}\;=\;r\;\cos\theta$$

where *θ_w_* is the apparent—measured and equilibrium and *θ* is the real—Young-contact angle. If the roughness of a surface is completely isotropic, then *R_a**S**_* = *R_a**T**_* and in consequence the apparent angles have the same value: cos *θ_w**T**_* = cos *θ_w**S**_*. But for an anisotropic surface in a complete wetting regime, it should be valid that only the real contact angels are the same: cos *θ**_T_*** = cos *θ**_S_***, and hence:

(4) $$\frac{r_{S}}{r_{T}}\;=\;\frac{\cos\theta_{wS}}{\cos\theta_{wT}}$$

Where *r**_S_*** and *r**_T_*** are the two-dimensional Wenzel factors—the length of the profile perimeters—along the ***S*** and ***T*** direction, respectively. For anisotropic surfaces it holds that *r**_T_*** > 1 and *r**_S_*** is the Wenzel factor of the whole surface, *r*. Thus,

(5) $$r\;=\;\frac{\cos\theta_{S}}{\cos\theta_{T}}$$

However, by applying Equation (5) to the available data no correlation was found, indicating that, although the angles are slightly lower than 90 degrees, complete wetting cannot be considered in all the interfacial areas.

**Figure 9:**
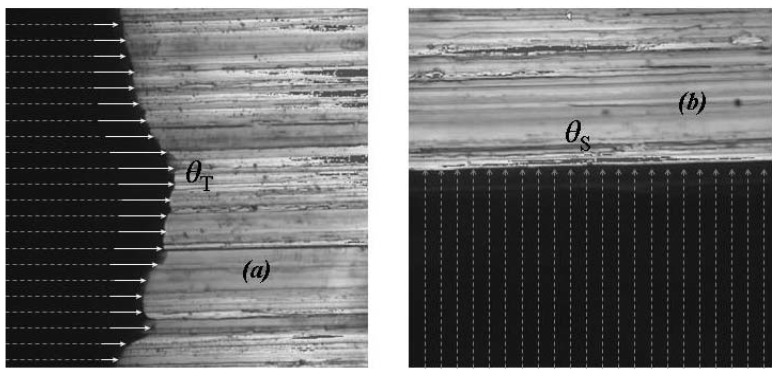
Complete wetting around the contact points where the angles were measured in ***T*** direction (***a***), Apparent contact angles measured in ***S*** direction correspond to a partial wetting regime (***b***). Measuring length 160 µm.

The apparent contact angles *θ_w_***_T_** measured in ***T*** direction at the position marked in Figure 9a are the smallest because, in these regions of the drop boundaries, the liquid is in complete contact with the surface. For this reason we can assume a complete wetting around the contact points where the angles were measured in ***T*** direction. On the contrary, the apparent contact angles measured in ***S*** direction correspond to a partial wetting regime.

As demonstrated above, if we consider partial wetting in the ***S*** direction but complete wetting on the drop boundaries in the ***T*** direction, it is possible to apply the Cassie and Baxter model [7,13] to the ***S*** direction. This model considers the wettability of a composite surface composed of two types of homogeneous patches that have different solid-fluid interfacial tensions. The apparent contact angle is then given by:

(6) $$\cos\theta_{cb}\;=f_{1}\;\cos\theta_{1}\;+\;f_{2}\cos\theta_{2}$$

where *f_i_* and *θ_i_* represent the surface area fraction and the contact angle of patch *i*, respectively. For porous or corrugated surfaces, the roughness is mainly filled with air. The openings of the pores can be regarded as nonwetting patches with *θ_2_* = 180°. Since *f*_2_ = 1 – *f*_1_, Equation (6) is:

(7) $$\cos\theta_{cbS}\;=f\;\left(\cos\theta \;+\;1\right)\;-\;1$$

where *f* is the quotient of the contact area surface and the projected area on the horizontal plane.

Equations (3) and (7) can be combined to obtain an expression to the solid fraction that considers the partial wetting along the ***S*** direction and complete wetting near to the boundaries of the ***T*** direction, as showed in Figure 9. Thus,

(8) $$f\;=\;\frac{r\;(\cos\theta_{cbS}\;+\;1)}{\cos\theta_{wT}\;+\;r}$$

**Figure 10:**
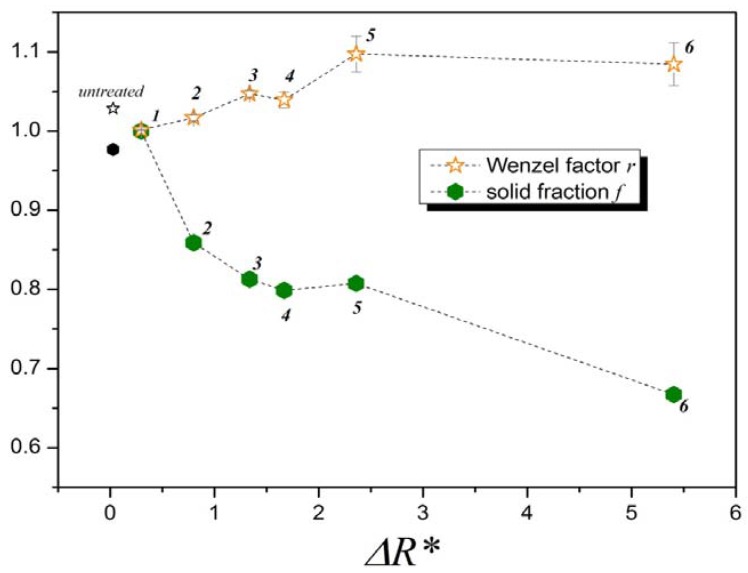
Dependence of Wenzel factor and solid fraction on surface anisotropy (ΔR*). The three parameters are dimensionless. Quantities on the Y-axis correspond to *r* and *f*. Errors are the standard deviations.

**Figure 11:**
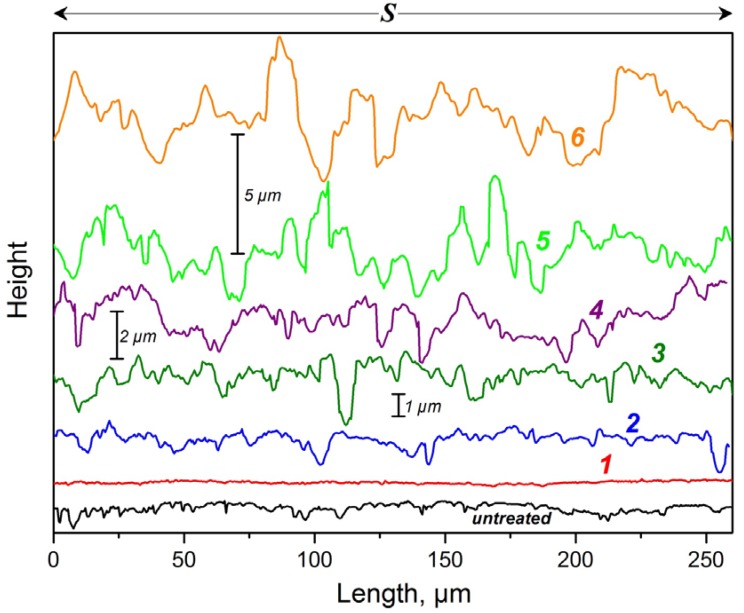
Comparison of the profiles of all samples.

The solid fractions can now be calculated using the static contact angles. Indeed, Figure 10 shows that the surface anisotropy (ΔR*) controls the partial wetting for Samples 2 to 6. Sample 1 presents, according to this model, almost complete wetting (*f = 1*). At the opposite end, Sample 6 is in the minimum of solid fraction, with only 67% of its surface being in contact with water. Figure 11 can help to better understand the morphological differences between the surfaces of the samples by comparing their profiles in ***S*** direction.

According to Figure 11, Samples 3 and 4 have similar profiles, therefore their Wenzel factors—*i.e.*, their perimeter profile lengths—shown in Figure 9 are almost the same. The profile of Sample 6 has higher peaks than that of Sample 5, but the distances between the peaks of Sample 6 are clearly larger and therefore its profile length—Wenzel factor—is relatively lower than that of Sample 5. Nevertheless Sample 6 has a higher topographical anisotropy (ΔR*)—difference between *R_a**T**_* and *R_a**S**_*—than Sample 5 (see Figure 10).

**Figure 12:**
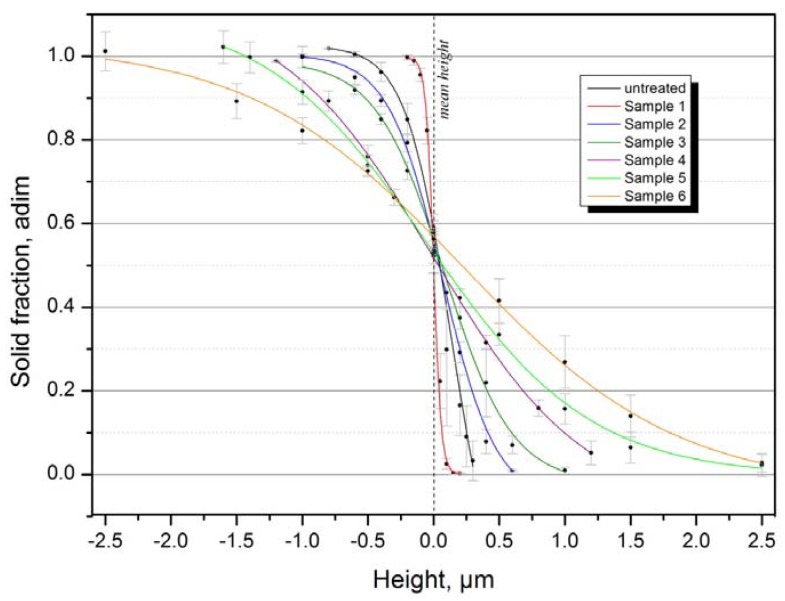
The sigmoidal “DoseResp” correlation between topography and solid fraction. Error bars are the standard deviations.

With the solid fraction values obtained using Equation (8), it is possible to estimate the size of the air volumes trapped in the interface of the surface and the water droplet. But for this purpose it is necessary to construct the curves “solid fraction *vs.* height”. The topographical data can be used as input to calculate the solid fraction *f* at different height levels using the software FRT Mark III (v.3.8.10). Using this procedure, we constructed the curves *h* *→ f*, where *h* is the height level with respect to the mean height (Figure 12). A very good function to correlate the points obtained is the sigmoidal “DoseResp” curve (Origin software v.8.61) that provides *R^2^* coefficients from 0.999 to 1:

(9) $$f(h)\;=\;a\;+\;\frac{b-a}{1+10^{(\log c-h)p}}$$

where *a*, *b*, *c* and *p* are correlation constants. Results are listed in Table 2.

**Table 2:** Sigmoidal correlation constants and the calculated interfacial air height with respect to the mean height plane. Errors of contact angles are the standard deviations.

Sample	*θ*_S_ (deg)	*θ*_T_ (deg)	*r* (adim)	*f* (adim)	*a* (µm)	*b* (µm)	*log c* (µm)	*p* (adim)	*R^2^* (adim)	h* (µm)
0	74.5 ± 4.0	72.7 ± 3.5	1.028	0.9768	−0.355	1.025	0.131	−2.535	0.999	−0.462
1	86.0 ± 1.3	91.7 ± 2.2	1.002	1	0.00061	0.997	−0.00036	−14.693	1	-
2	84.5 ± 7.8	73.9 ± 3.4	1.017	0.8588	−0.065	1.004	0.066	−2.124	1	−0.317
3	82.5 ± 7.9	67.0 ± 1.3	1.039	0.8127	−0.021	0.99	0.079	−1.685	0.999	−0.336
4	81.5 ± 7.0	64.1 ± 7.4	1.047	0.7989	−0.085	1.127	−0.005	−0.745	1	−0.531
5	88.8 ± 7.5	74.7 ± 4.3	1.098	0.8076	−0.003	1.108	−0.046	−0.698	0.999	−0.708
6	95.4 ± 9.5	69.0 ± 1.0	1.085	0.6670	−0.034	1.029	0.222	−0.533	0.998	−0.365

**Figure 13:**
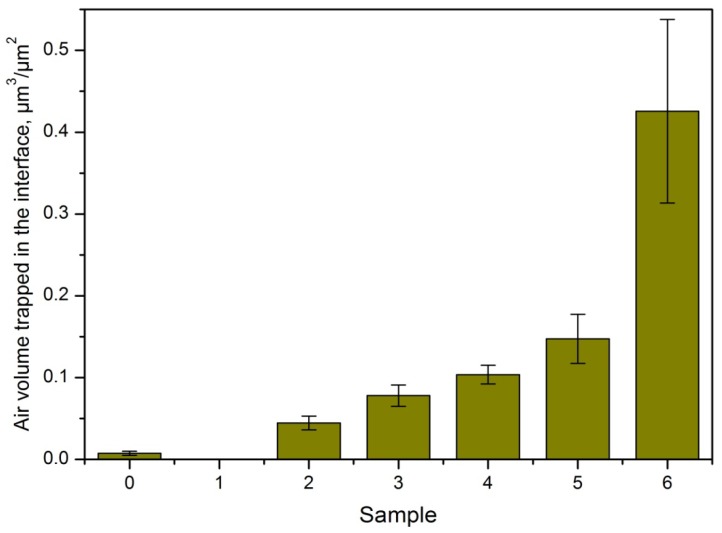
Calculated air volume trapped in the interface stainless steel-water. Errors are the standard deviations.

**Figure 14:**
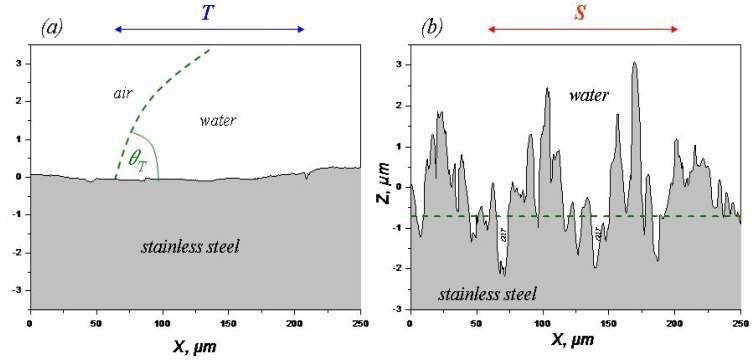
2D-profiles and the calculated air interface height for Sample 5 using the topographical data. Complete wetting in the direction parallel to the grooves (**a**), and partial wetting (**b**) in the direction perpendicular to the grooves with a calculated solid fraction at *h* = −0.708 µm (Table 2).

By interpolating the solid fraction sigmoidals of Figure 12 with the solid fractions reported in Figure 10 it is possible to obtain the height of the interfacial air of each sample. Using again the software FRT Mark III (v. 3.8.10) it was possible to calculate the air volume trapped in the interfaces (Figure 13), *i.e*., the void volume between an imaginary plane at height *h* and the surface of the sample. Finally, using the topographical data, it is possible to represent the profile and the air interface height of the surfaces investigated as shown Figure 14 for Sample 5.

## 5. Conclusions

The effect of topographical anisotropy of a stainless steel surface on water contact angle was investigated. The correlation between topography and wetting can be described by the T-S Model proposed in the present work. This model, which can be summarized by Equation 8, links the contact angle information measured in both perpendicular directions and the Wenzel roughness as well as the solid fraction. Using this model it was possible, independent of the relative hydrophilic surface of the steel (static contact angles <90°), to show that the wetting on the steel surface was not complete due to the air present in the deep cavities of the surface.

**Figure 15:**
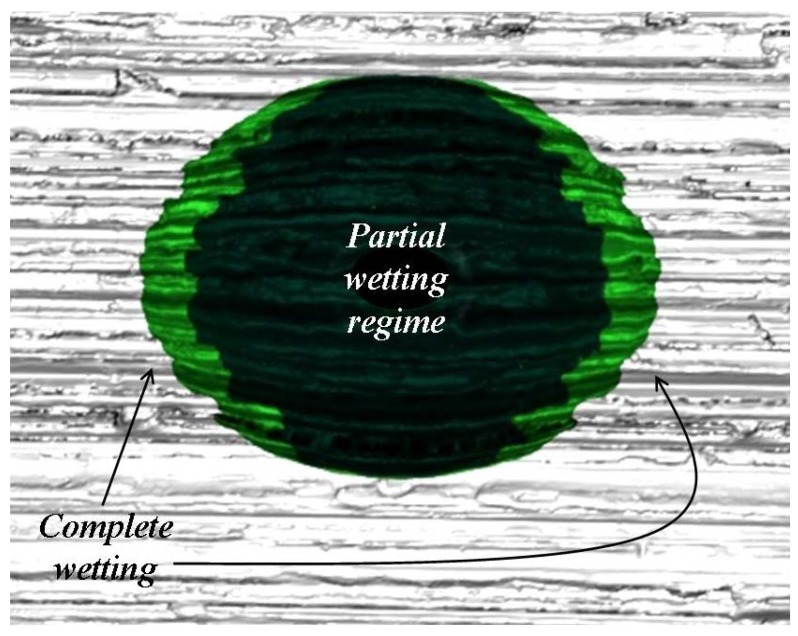
Complete wetting around the contact points where the angles were measured in ***T*** direction (green regions). The apparent contact angles measured in ***S*** direction correspond to a partial wetting regime (dark regions).

The apparent contact angle *θ**_T_*** measured in ***T*** direction (parallel to grooves direction) is the smallest possible for the ground samples because in these regions of the drop boundaries, the liquid is in complete contact with the bottom surface of the grooves. For this reason we can conclude that wetting is complete around the contact points where the angles were measured in ***T*** direction (green regions in Figure 15). On the contrary, the apparent contact angles measured in ***S*** direction (perpendicular to grooves direction) correspond to a partial wetting regime (dark regions in Figure 15).

According to the above, our model is a validation of that presented by Kioshi *et al* [5] for the molecular scale to the microscopic scale (see Section 1). These authors suggested the existence of transition conditions between Wenzel and Cassie-Baxter regimes, which was experimentally confirmed in this study. Finally, a further application of the model presented in this article is to quantify the air present between solid and liquid phases using the topographic information and the measurements of contact angle anisotropy.
